# Increased risk of coronary perforation during percutaneous intervention of myocardial bridge: What histopathology says

**DOI:** 10.15171/jcvtr.2017.18

**Published:** 2017-06-29

**Authors:** Somayeh Pourhoseini, Mohammad Bakhtiari, Abdolreza Babaee, Mohammad Ali Ostovan, Seyed Hassan Eftekhar-Vaghefi, Nikan Ostovan, Pooyan Dehghani

**Affiliations:** ^1^Department of Anatomy, School of Medicine, Kerman University of Medical Sciences, Kerman, Iran; ^2^Department of Anatomical Science and Molecular Biology, Isfahan University of Medical Sciences, Isfahan, Iran; ^3^Department of Cardiology, School of Medicine, Shiraz University of Medical Sciences, Shiraz, Iran; ^4^Shiraz Cardiovascular Research Center, Shiraz University of Medical Sciences, Shiraz, Iran

**Keywords:** Myocardial Bridge, Coronary Perforation, Histopathology, Percutaneous Coronary Intervention

## Abstract

***Introduction:*** Myocardial bridge (MB) is a segment of a major epicardial coronary artery that goes intramurally under a bridge of overlying myocardium. Complications have been reported during or after stent implantation particularly coronary perforation. The aim of this study was to determine histological differences between proximal left anterior descending artery (LAD) and the tunneled segment that may have a possible role in increased risk of coronary artery perforation during percutaneous coronary intervention.

***Methods:*** Twenty specimens of MB were obtained from dissection of 45 cadavers. Sections were stained using hematoxylin and eosin (H&E), and trichrome methods. The proximal section and the tunneled artery were compared with a normal sample in terms of the characteristics of a muscle artery.

***Results:*** The findings of this study showed an MB prevalence of 51%, as 23 out of the 45 examined cadavers were discovered to be afflicted by the MB. The intima layer in the suffering artery had gone through significant hypertrophy, while it had remained thin in the tunneled artery section. The epithelial cells under the bridge were spindle-shaped, while they were polygonal in the proximal section. In the myocardium the nuclei of the muscle fibers in the MB section were smaller than the normal section. Adventitial layer was almost normal.

***Conclusion:*** The histopathological differences between MB and proximal part of vessel combined with small vessel diameter in the tunneled segment can explain the high incidence of the LAD rupture and perforation in the section under the bridge.

## Introduction

Myocardial bridge (MB), an inborn coronary abnormality,^1,2^ is defined as a segment of a major epicardial coronary artery, the tunneled artery, that goes intramurally under a bridge of overlying myocardium.^3^ MB is generally confined to the left anterior descending artery (LAD).^4^ Anatomically, MB is classified as superficial or deep depending on its width. The length of a typical MB is usually within 10 to 30 mm range, only rarely exceeding 40 mm.^5^ It is a common coronary disorder with an average prevalence of (30%) in general population,^6,7^ which, however varies substantially among studies, with a much higher rate at autopsy than angiography.^7^

Traditionally, myocardial bridging has been considered a benign condition, but symptoms such as angina-like chest pain have been reported. Also, various studies have revealed an association between myocardial bridging and sudden cardiac death,^8^ myocardial infarction,^9^ arrhythmias,^10^ and myocardial ischemia.^11^

In symptomatic MB cases, the occurrence of coronary artery disease is considered to be caused by the direct MB compression of the LAD.^12^ The segment proximal to the bridge frequently shows atherosclerotic plaque formation, although the tunneled segment is typically spared.^2^ Besides, the likelihood of ischemia increases with the intra-myocardial depth of the tunneled segment.^12^

With regard to the treatment, three strategies have been explored: (1) Negative inotropic and/or negative chronotropic agents i.e. beta blockers and calcium antagonists,^11,13^ (2) Surgical myotomy and/or coronary artery bypass graft surgery (CABG),^11,14^ (3) Stenting of the tunneled segment.^11,15,16^

Percutaneous coronary intervention (PCI) with stent implantation under the MB is used mainly in patients with severe systolic and diastolic stenosis, complete occlusion at the bridged segment, resistance to drug therapies or when there is concomitant atherosclerotic lesion near the muscle bridge. Complications have been reported during or after stent implantation particularly coronary perforation during or immediately after stenting.^16^ In our own experience we had eight cases of PCI on muscle bridge segments that were complicated with coronary perforations.

The increased risk of perforation during PCI can be due to multiple factors. Thin intima and a probable smaller vessel diameter of the tunneled segment are said to be two possible causes. Over inflation of the balloon and oversizing of the stent could be another mechanism leading to coronary rupture.^17^

The purpose of our study was to determine histological differences between proximal (LAD) and tunneled artery that may have a possible causative role in increased risk of coronary artery perforating during PCI.

## Materials and Methods

### Study design

To determine the prevalence of MB and its histological features a collection of 20 cases were needed based on Chocran’s sample size formula with 95% confidence interval and alpha level of 0.05. In other words, to come up with a reliable outcome the required sample volume was 20 cases of MB. To get access to the target sample volume of 20, 45 cadavers, from Shiraz Forensic Center were examined. The sample consisted of 24 males and 21 females in the age range between 17 and 80. Out of the 45 examined cadavers, 23 were discovered to suffer from MB. None of the 23 spotted cases had a record of heart complaints and their death had occurred due to other reasons. In line with the statistical advisory for a sample volume of 20, only 20 out of the 23 detected MB cases went through the tissue processing procedure.

### Tissue sampling procedures

To collect the required tissue, from the supraclavicular area to the pubic symphysis, the skin was incised and the thorax was pressed aside to be able to remove the heart from the epicardium. This procedure was performed very accurately, observing all required anatomical techniques.

**Figure 1:**
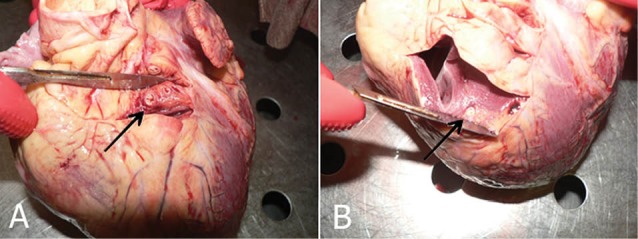
A, B) The tunneled artery, that goes intramurally through myocardium (The arrow).

In the next stage, the heart was closely examined and using traverse incisions, the LAD artery was followed in the ventricular groove to the apex. Samples were taken from the proximal and tunneled artery of the MB cases (Figure 1).

### Tissue processing procedure

#### Fixation procedure

The samples were kept in the fixative solution (10% formalin) for at least 7 days. Following this, using LECAT POLO, the samples went through tissue passaging procedure of dehydration, clearing, and impregnation for 12-16 hours.

In the embedding stage, samples were vertically placed inside metal frames, and covered in melted paraffin to cool down to paraffin blocks which were kept in the refrigerator prior to sectioning.

Microtome MICROM HM 235 was used to prepare 5 micro centimeter traverse sections which were fixed on slides, labeled with the samples’ information, and heated in the oven to melt the extra paraffin.

#### Staining procedure

In the staining stage, the sections were stained using hematoxylin and eosin (H&E), and trichrome methods.

For histological studies, the stained slides were studied using optic microscope equipped with camera and lens. In the investigation process, the proximal section and the tunneled artery were compared with a normal sample in terms of the characteristics of a muscle artery.

## Results

Concerning the prevalence rate of MB in general population, the findings of this study illustrated that out of 45 cadavers examined for evidence of MB, 23 were afflicted with MB, resulting in a prevalence of 51%. For tissue processing procedure, however, only 20 MB cases were selected in line with the recommendation of the statistical adviser of the project for a sample volume of 20 MB cases.

**Figure 2:**
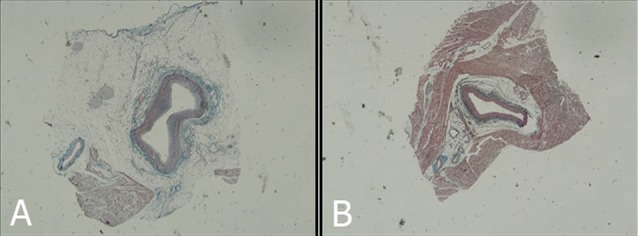
The comparisons between proximal (A) and tunneled artery in intimal layer

With regard to the histological findings, the histological comparisons between MB samples and normal specimens revealed that in both the proximal and the tunneled artery, the intima layer had suffered certain transformations as the intima layer in the proximal section showed remarkable hypertrophy, while in the tunneled artery section, the intima layer was thinner compared with the proximal layer, resulting in a remarkable difference between the two regions, in terms of their sizes (Table 1; Figure 2).

**Table 1:** Intimal diameters of the bridged artery (µm)

	**Mean**±**SD**	**Median (min-max)**	***P*** ** value**
Intimal diameter of proximal part (µm)	397.13±55.74	417.27 (300.44-459.47)	0.005*
Intimal diameter of tunneled segment (µm)	62.85±2.56	62.92 (58.55-66.28)	
Intimal diameter difference between proximal and tunneled part (µm)	334.28±56.32		

In the proximal artery section, the majority of endothelial cells of the MB samples were spiral-shaped, while in the tunneled artery section, the endothelial cells were spindle shaped.

**Figure 3:**
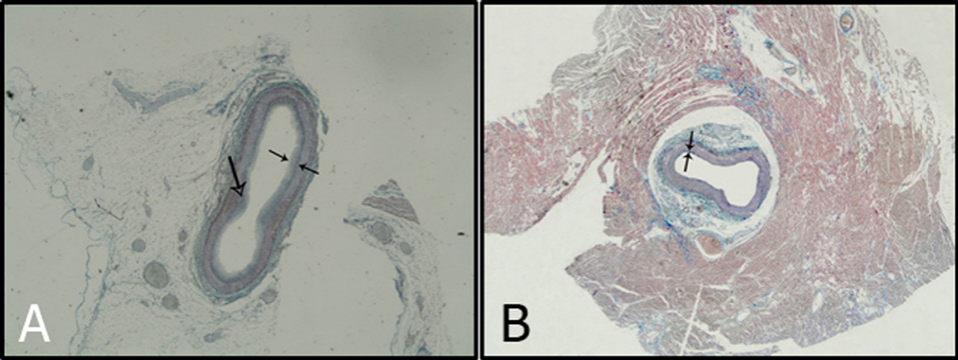
(A) Intimal layer of a proximal part of myocardial bridge with atherosclerosis plaque (Large arrow). (B) Intimal layer of tunneled segment. Note the difference of diameters.

In the majority of samples, atherosclerotic plaques were observed in the proximal artery section, while in the tunneled artery section of the samples, no plaques were observed. The Adventitial layer was almost normal (Figure 3).

Concerning the myocardium, in the MB section, it was significantly different from the rest of the myocardium, as the nuclei in the muscle fibers of this section were smaller than the normal sections.

## Discussion

The major purpose of the histological examination of the present study was to find an explanation for the high incidence of coronary perforation during or following the stenting of the bridged segment of the coronary artery which is reported in literature.^17-21^

In our own practice we had also eight cases of coronary rupture of the bridged part during PCI. The mean age of our cases was 59.6 years including 7 females and 1 male. Five cases were complicated with cardiac tamponade needing pericardiocentesis and pigtail insertion. Three developed with intracavitary perforations with no evidence of tamponade.

**Figure 4:**
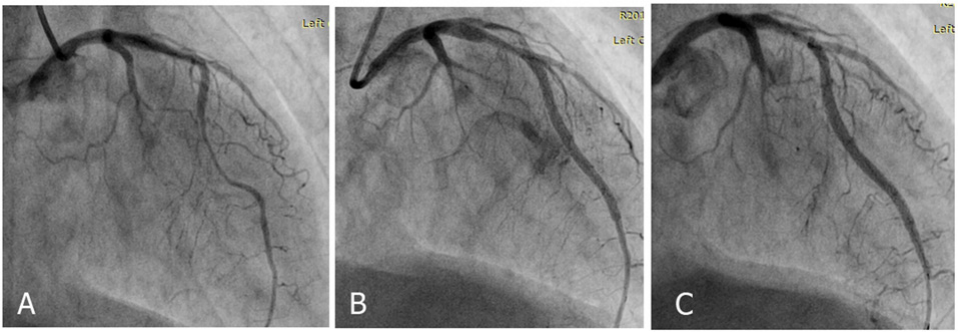
Coronary angiography shows long segment muscle bridge (A), Perforation after stent implantation (B), sealing of perforation after multiple prolonged balloon inflation (C).

**Figure 5:**
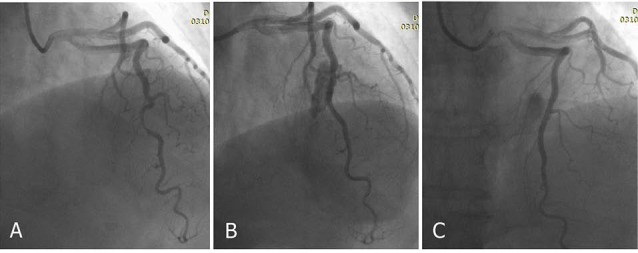
Coronary angiography shows significant compression of muscle bridge segment (A), Perforation and suspected proximal stent edge dissection after stenting (B), sealed perforation after Bare metal stent implantation and intermittent prolong balloon inflation (C).

**Figure 6:**
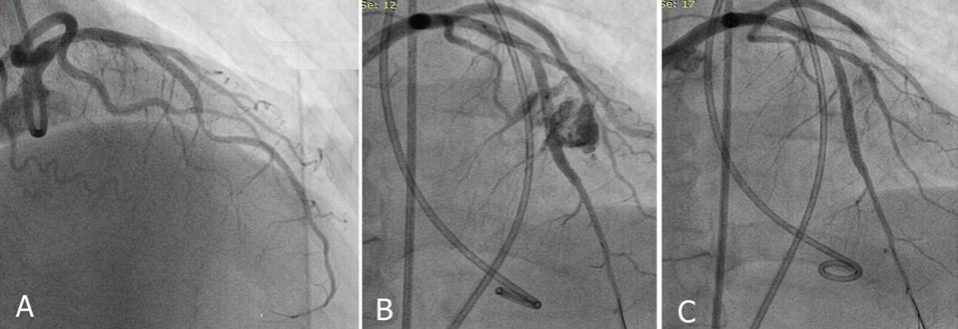
Coronary angiography reveals significant muscle bridge (A), perforation of vessel after stent implantation with pigtail insertion (B), sealing of perforation after deployment of covered stent (C).

In 2 cases, the perforation was sealed with multiple prolonged balloon inflations (Figure 4). In the other, a bare metal stent was implanted because of suspicion of perforation due to edge dissection combined with repeated longstanding balloon inflations (Figure 5). We had to seal the perforation with covered stents, in the remaining five (Figure 6).

In 2008, Li et al, reported 2 cases of perforations, one was sealed with prolonged balloon inflations and the other was sent for emergency cardiac surgery due to no response to the aforementioned management.^20^ Shen et al presented a case of coronary perforation during PCI for muscle bridge segment, successfully managed with implanting covered stent.^21^

In an interesting study by Haager et al,^22^ long term follow up of patients who underwent PCI for symptomatic myocardial bridging was investigated. They presented 11 cases out of which 4 developed with significant in stent restenosis. They concluded that high inflation pressures may be needed for optimal stent implantation and apposition. Intravascular ultrasound (IVUS) is helpful to achieve the favorable result. One of our cases with coronary perforations, developed with significant in stent restenosis after 1 year which was sent for coronary artery bypass graft surgery.

This increased risk of perforation during PCI is actually a multifactorial phenomenon. Thin intima, smaller vessel diameter of the tunneled segment, over inflation of the balloon and oversizing of the stent are some probable causes.^17,23^ To this purpose, histological differences in the proximal and tunneled artery were examined. The findings of this study showed an MB prevalence of 51%, as 23 out of the 45 examined cadavers were discovered to be afflicted by the MB. Different rates of MB prevalence has been reported in various studies, with a higher percentage reported in autopsies than conventional and even CT angiographic studies.^7^ This variety can be explained by the fact that CT angiography is not capable of detecting MBs thinner than 20 mm, and such MBs can be diagnosed only through autopsy.^24^ The prevalence of MB through autopsy is 50%-58%, indicating the highest incidence rate of MB^4^ compared to other techniques. For instance, following autopsy examining of 90 cadavers, Ferreira et al^4^ reported detecting MB in 55 cases, giving a prevalence of almost 55%, in people without any previous history of heart complaints, whose death had occurred due to reasons other than heart problems. Consequently, the findings of the present study in this regard is compatible with similar studies.

Our study showed that the intima layer in the suffering artery had gone through significant hypertrophy, while it had remained thin in the tunneled artery section. Previous studies confirm these findings.^4,6^ It has been reported that the intima layer has different characteristics before, below and after the bridge. Before the bridge, the intima layer has been reported to be about 406.6 µm wide on average, while under the bridge its width is about 66 µm.^6^

Furthermore, the present study found that the epithelial cells under the bridge were spindle-shaped, while they were polygonal in the proximal section. Previous studies had also reported that the endothelial cells had a helical orientation (associated with the laminar blood flow and high endothelial shear stress) under the bridge, and polymorph, flat, or polygonal shapes before the bridge (shapes associated with low endothelial shear stress).^6,24^

We also observed that in the myocardium the nuclei of the muscle fibers in the MB section were smaller than the normal section, an observation reported by other studies, too.^4,6,24^ There seems to be significant differences within the myocardial structure between samples taken from the bridge areas vascularized by tunneled coronaries, and the rest of the myocardium. The nuclei from the MB section fibers are always smaller than the ones from other areas.^25^ Furthermore, through comparing MB hearts with non-MB hearts, Brodsky found a significantly increased interstitial fibrosis in samples obtained from the anterior wall of the left ventricle as compared with equivalent samples from cases without MB.^26^

## Conclusion

Based on the aforementioned discussion, it can be concluded that MB structure varies from the rest of the myocardium in that the nuclei from the bridged myocardial fibers are smaller and interstitial fibrosis is higher in this area. Besides, the bridge makes transportation in the intima structure. These three factors can result in the over-contraction of myocardium in this section, besides inhibiting the full extension of the artery. The latter and smaller vessel diameter in the tunneled segment can explain the high incidence of the LAD rupture and perforation in the section under the bridge.

## Competing interests

The authors declare that they have no competing interests.

## Ethical Approval

This study was approved by ethics committee, Shiraz University of Medical Sciences, Shiraz, Iran.
